# An intersectional approach to identifying factors associated with anxiety and depression following the COVID-19 pandemic

**DOI:** 10.1038/s41598-022-15695-5

**Published:** 2022-07-06

**Authors:** Hoda Seens, Ze Lu, James Fraser, Joy C. MacDermid, David M. Walton, Ruby Grewal

**Affiliations:** 1 Health and Rehabilitation Sciences, Western University, London, ON Canada; 2 Windsor University School of Medicine, Cayon, Saint Kitts and Nevis; 3 School of Rehabilitation Science, McMaster University, Hamilton, ON Canada; 4 Roth McFarlane Hand and Upper Limb Centre, St. Joseph’s Health Care London, London, ON Canada; 5 School of Physical Therapy, Western University, London, ON Canada; 6 Department of Computer Science, University of Guelph, Guelph, ON Canada; 7 Schulich School of Medicine and Dentistry, Western University, London, ON Canada

**Keywords:** Psychology, Health occupations, Medical research, Risk factors, Signs and symptoms

## Abstract

The COVID-19 pandemic is impacting mental health, with some populations bearing a greater burden. In this cross-sectional online study, we examined the personal and intersectional factors associated with increased symptoms of anxiety and depression following the COVID-19 pandemic. We assessed pre- and post-pandemic levels of anxiety and depressive symptoms using the Generalized Anxiety Disorder-2 (GAD-2) and Patient Health Questionnaire-9 (PHQ-9) scales, respectively. The study included 1847 participants, with an age range of 18 to 79 years and representing 43 countries. Variables with significance (*p* < 0.05) in predicting post-pandemic GAD-2 and PHQ-9 scores were pre-pandemic scores on the same scales, an interaction between increasing age and non-man gender, and an interaction between non-man gender and having children. Health practitioners, psychiatrists, and policy makers need to be aware and respond to the mental health burden of the pandemic on women and other gendered individuals, especially those who care for children.

## Introduction

Cases of the novel coronavirus (2019-nCoV) were first reported on December 29, 2019 as a “pneumonia of unknown etiology”^1^. The World Health Organization called this new coronavirus “COVID-19” and deemed it a pandemic on March 11, 2020^2^. Early in the pandemic, researchers demonstrated the negative mental health consequences and psychological distress it was having on populations^3–6^.

Similar to its varied physical effects on individuals and communities^7^, the virus and pandemic affect the mental health of individuals and groups in differing ways. For example, women and females have experienced greater levels of anxiety and depression than men and males^8–11^. Another widely examined relationship is age and mental health during the pandemic. One study, conducted in China, found that younger adults (ages 18 to 30 years) experienced greater levels of psychological distress^10^. An Italian study found greater stress levels among young people working outside of the home^9^. A Canadian analysis determined an increased likelihood of anxiety and depression among the younger segments of the population^11^. Another Canadian study found increased levels of anxiety and poor self-perceived mental health among those who were 15 to 24 years of age, with decreasing levels of anxiety as age increased, until the age of 65 years at which point anxiety and poor self-perception of mental health increased again^12^. A study of an American population found decreasing levels of anxiety and depression among older adults^13^.

The impact of the pandemic has been examined in relationship to other individual variables, such as education levels, previous morbidities, and healthcare occupation. Those with higher levels of education, such as post-secondary education or above, have experienced greater psychological distress during the pandemic^10,14^. Those with pre-existing medical conditions have reported greater levels of anxiety and depression during the COVID-19 pandemic^9^. There have also been greater levels of distress reported among healthcare workers^14^, and increased levels of anxiety reported among physicians^15^. One study found increased levels of anxiety among family members of healthcare workers, especially those healthcare workers in direct contact with COVID-19 patients^16^.

## Work and family life during the pandemic

In addition to the demographic variables associated with psychological distress during the COVID-19 pandemic, personal and life circumstances can impact the levels of distress that individuals are experiencing. With changes in patterns of work, wherein some previously-employed workers have lost their jobs, others began working from home, and some undertook greater risk to their personal health, an individual’s work and work location can have an impact on psychological distress, including levels of anxiety and depression^17–19^. Likewise, numerous jurisdictions worldwide closed in-person schools for children in efforts to mitigate the spread of the virus. School closures left many parents caring for their children at home, home-schooling, and/or working from home while also caring for school-aged children. This led to increased reported levels of caregiver distress and parental burnout^5,20^.

## Intersectionality in mental health

The term “intersectionality” was first coined by Kimberlé Crenshaw in 1989 to refer to the interplay between a person’s various identities, which are influenced to different extents by dominance and oppression^21^. Intersectionality moves beyond individual factors (such as gender, age, and race) to examine the interaction between these factors, and assess how the health of populations are shaped^22^. Despite the complexities of the determinants of an individual’s mental health, research about intersectionality in mental health is limited and methodologically unstandardized^23^. In our study, we endeavored to examine mental health holistically by taking into account the interplay between various identifies.

## Study objectives

Our first objective was to understand the personal factors that are correlated with post-COVID-19 levels of anxiety and depressive symptoms. Our second objective was to investigate how the post-pandemic levels of anxiety and depression have been affected by intersectional factors, namely the interactions between age and gender, age and marital status, gender and having a child(ren), gender and working from home, and working from home and having a child(ren). We used March 11, 2020 as the beginning of the COVID-19 pandemic and refer to any date after March 11, 2020 as “post-pandemic.”

## Methods

### Study design

For transparency, we have adhered to recommendations set forth in *The Checklist for Reporting Results of Internet E-Surveys (CHERRIES)*^24^. Additionally, we aimed to follow *The Sex and Gender Equity in Research (SAGER)* guidelines^25^ to ensure that we included specific questions on sex and gender in our study design. We also remained cognizant of the importance of sex and gender factors throughout our analyses and the reporting of findings.

We developed the survey consisting of questions in the following categories: demographics, mental health, and homelife questions. We combined standard and previously validated scales with survey-specific questions. The survey was designed and administered in English on the Qualtrics platform version June-July–August, 2020 (Qualtrics, Provo, UT). After transferring the survey into the web platform, we ensured survey functionality through pre-testing by graduate students with clinical and methodologic expertise.

Western University’s Health Sciences Research Ethics Board approved this study (project identification number: 115790) on June 25, 2020. All respondents were asked to read through a letter of information describing the study and providing informed consent. Consent was the first question on the survey and its completion was required prior to the display of the survey questions. At the end of the survey, respondents were able to enter a draw to win one of three Amazon gift cards (35 USD each).

The survey questions were administered to all respondents in the same order. Some survey questions had adaptive parts and were conditionally displayed depending on a response to a previous item. Other than the question seeking informed consent, participants could leave blanks or skip items if they did not desire to give an answer. If they wished, respondents were also able to change answers to previous questions. Participants had up to two weeks to return and complete their responses. Qualtrics uses internet protocol addresses to prevent duplicate entries from respondents.

### Participants and data collection

Inclusion criteria for participants were being 18 years of age or older and able to read and respond in English. As described above, participants had to provide informed consent to the letter of information to be included in the study. As with other online surveys completed from home, participants had to use a device with internet access. Respondent anonymity was maintained throughout the study.

We used various online channels to recruit participants. We used community websites (Kijiji and Craigslist), social media sites (Facebook, Instagram, and Twitter), and Whatsapp groups and email listservs. We began data collection on June 26, 2020 and closed the survey on August 31, 2020. Therefore, data was collected for approximately ten weeks.

### Measures of independent variables

#### Demographics

The survey began with a set of demographic questions. First, we asked respondents to state where they lived by picking their country and state/province from a drop-down menu and writing in the name of their city of residence. Next, we asked about respondents’ current employment status, marital status, and the number of individuals living in their homes (including the number of dependent children). Age, biological sex, gender identity, and ethnic origin(s) were then collected by selection from pre-defined fields, with an “other” option for sex, gender, and ethnicity.

#### Marital, child, and work status

In terms of their marital status, the survey asked respondents to choose between single, common-law, married, divorced, widowed, and other in response to the question: “What is your marital status?” For having children, participants were asked “how many children (who are dependent on you) currently live in your home?” Respondents were presented with a drop-down menu ranging from 0 children to more than 10, in increments of 1 child.

For work status, we asked, “Which statement best describes your current employment status?” Participants could choose between paid employee, self-employed, laid off, stay-at-home parent/caretaker, student, retired, unable to work due to a disability, and other. If respondents answered that they were a paid employee or self-employed, they would be presented with a follow-up question asking them to write their job title. If respondents chose that they were laid off, they would be asked to write in what their job used to be. If respondents answered that they were students, they were asked to write what they study. Then respondents were asked about changes to their job status as a result of the pandemic.

Finally, respondents were asked if “As a result of the pandemic, do you have to work from home?” The options were “with a similar workload” (1), “with a greater workload” (2), “with a lighter workload” (3), “I still go into my work place” (4), “I lost my job” (5), and “I have always worked from home” (6). In our analysis of workplace location, we categorized working from home as a response to items 1, 2, 3, or 6; working outside of the home is a response to item 4; and, job loss is a response to item 5.

### Measures of dependent variables

#### Anxiety symptoms

Respondents were asked to complete two copies of the Generalized Anxiety Disorder-2 (GAD-2) scale corresponding to their pre- and post-pandemic states. We asked respondents to fill the first copy to represent an average two-week period before the start of the pandemic (March 11, 2020) and the second copy to represent an average two-week period after the start of the COVID-19 pandemic. That is, respondents were asked to recall their average state for both the pre- and post-COVID-19 timepoints.

GAD-2 is a patient-reported tool for screening generalized anxiety disorder (GAD)^26^ with two items where respondents answer “not at all,” “several days,” “more than half the days,” and “nearly every day” to each item. The items are then rated on a four-point Likert scale (0 to 3), which is added together to arrive at a total score (ranges between 0 and 6)^27^. A total score of 3 or greater indicates a clinically relevant anxiety disorder and has a sensitivity and specificity of 86% and 83%, respectively^26^. Tests of reliability for GAD-2 demonstrated internal consistency of 0.81 (Cronbach’s alpha) and test–retest reliability 0.81^28^.

#### Depressive symptoms

We asked respondents to also complete two copies of the Patient Health Questionnaire-9 (PHQ-9) representing their pre- and post-pandemic states. Similar to GAD-2, participants were asked to fill the first copy to represent an average two-week period before the start of the pandemic (March 11, 2020) and the second copy to represent an average two-week period after the start of the COVID-19 pandemic.

The PHQ-9 is a screening tool for major depressive disorder (MDD), and may also be used to diagnose MDD or measure its severity^28^. Similar to GAD-2, respondents answer all questions choosing “not at all,” “several days,” “more than half the days,” and “nearly every day.” On a four-point Likert scale (0 to 3), the responses on the questionnaire are summed (ranges between 0 and 27)^28^. Total PHQ-9 scores can be used to denote MDD severity with 5, 10, 15, and 20 indicating mild, moderate, moderately severe, and severe depression, respectively. For scores between 5 and 9, it is recommended that individuals are monitored for changing levels of depressive symptoms^29^. When the PHQ-9 score is 10 or greater, the scale’s sensitivity and specificity are both 88%^29^. Tests of reliability for PHQ-9 demonstrated internal consistency of 0.89 (Cronbach’s alpha) and test–retest reliability 0.84^30^.

### Statistical analysis

We performed hierarchical (stepwise) multiple linear regression to determine the optimal regression model for both GAD-2 and PHQ-9 post-pandemic states. We did this in three steps by inserting the following into the two models: (1) personal demographic factors, (2) personal demographic factors and working location, and (3) personal demographic factors, working location, and conjunct variables.

Personal demographic variables that we considered in the model were: country of residence, gender (man, woman, non-binary, agender, or other), marital status (single, common-law, married, divorced, widowed, or other), having a child(ren) (yes or no), age, and pre-pandemic mental health scores (either GAD-2 or PHQ-9). Working location factors that we considered were: work from home, still go to work, or lost one’s job. The variables of country, gender, marital status, having a child(ren), and working location were treated as dummy variables. That is, they were categorical variables with no meaningful order. Age (years) and pre-pandemic mental health scores (GAD-2 and PHQ-9) were continuous variables in the models. As part of step 3, we created conjunct variables to further explore the interactions between personal indicators. We created the following conjunct variables: age × gender, age × marital status, gender × child (yes/no), gender × working location, and working location × child.

To investigate all the potential independent variables, the significant level for entrance into the stepwise model was set at p < 0.05; while for removal, it was set as p > 0.10. Regression diagnostics were performed to check the assumptions of multivariate linear regression. As part of the assumption validation process, the variance inflation check indicated that there was no collinearity in the independent variables. The assumption of constant variance across the independent variables was also confirmed using the Breusch-Pagan test. All statistical tests were 2-tailed, and an effect was considered significant if p < 0.05. All analysis was performed by IBM SPSS statistics, Version 25.0 (IBM Corporation, Armonk, NY).

### Ethical approval

Ethical approval was obtained from Western University’s Health Sciences Research Ethics Board on June 25, 2020 (project identification number: 115790). All methods were carried out in accordance with the Helsinki declaration and its subsequent amendments. Participants were asked to read an online letter of information and provide written consent by choosing “Yes, I consent to participate” or “No, I do not consent.” If participants (18 years of age and older) provided their informed consent, study questions were displayed to begin the survey.

## Results

### Demographic characteristics

The overall study included 1847 consenting participants, of whom 1397 respondents (75.6%) completed the survey. In the analysis of this study, we have included the subset of participants who have complete data for pre- and post-pandemic GAD-2 (n = 1379) and PHQ-9 (n = 1287) scales. The age range for participants is 18 to 79 years, with a mean of 30.3 (± 13.3) years for GAD-2 participants and 30.4 (± 13.5) years for PHQ-9 participants, respectively, and includes 43 countries of residence (as displayed in Table 1). The gender representation for man, woman, and other genders (non-binary, agender, and other) for GAD-2 respondents who reported gender is 283 (20.6%), 1058 (77.1%), and 31 (2.6%), respectively; and for PHQ-9 respondents who reported gender is 268 (20.9%), 984 (76.9%), and 28 (2.2%), respectively.

**Table 1:** Demographic characteristics.

Demographic	GAD-2 n (%)/mean (SD)	PHQ-9 n (%)/mean (SD)
**Sex**	1373	1281
Male	289 (21.0%)	273(78.1%)
Female	1076 (78.4%)	1000(21.3%)
Other	8(0.6%)	8(0.6%)
**Gender**	1372	1280
Man	283 (20.6%)	268(20.9%)
Woman	1058(77.1%)	984(76.9%)
Others^a^	31 (2.2%)	28(2.2%)
**Age**	1367	1276
Mean (SD)	30.3 (13.3) years	30.4 (13.5) years
**Location (country/continent**^b^**)**	1379	1287
Canada	1023 (74.2%)	951(73.9%)
USA	264(19.1%)	248(19.3%)
Europe	35(2.5%)	33(2.6%)
Asia	35(2.5%)	33(2.6%)
Americas	11(0.8%)	11(0.9%)
Oceania	7(0.5%)	7(0.5%)
Africa	4(0.3%)	4(0.3%)
**Marital status**	1347	1257
Single	838 (62.2%)	778(61.9%)
Common-law	84(6.2%)	77(6.1%)
Married	368(27.3%)	350(27.8%)
Divorced	50(3.7%)	45(3.6%)
Widowed	7(0.5%)	7(0.6%)
**Child(ren)**	1370	1280
Yes	358(26.1%)	342(26.7%)
No	1012(73.9%)	938(73.3%)
**Working location**	696	644
At home	376(54.0%)	353(54.8%)
Outside of home	304(43.7%)	276(42.9%)
Lost job	16(2.3%)	15(2.3%)

^a^Other genders includes participants who responded: non-binary, agender, and other.

^b^Europe (Croatia, France, Germany, Ireland, Italy, Netherlands, Romania, Slovakia, Spain, Sweden, Switzerland, United Kingdom), Asia (China, India, Iran, Israel, Japan, Kazakhstan, Malaysia, Oman, Pakistan, Philippines, Qatar, Saudi Arabia, Singapore, United Arab Emirates), Americas (Antigua and Barbuda, Argentina, Bahamas, Honduras, Jamaica, Peru, Saint Kitts and Nevis), Oceania (Australia, New Zealand), Africa (Ethiopia, Namibia, Nigeria, South Africa).

### Anxiety symptoms

The mean pre-pandemic GAD-2 score was 2.1 (95% confidence interval (CI) 2.0 to 2.2) and increased to 3.3 (95% CI 3.2 to 3.4) following the pandemic (after March 11, 2020). This change represents a mean difference of 1.2 (out of a possible 6 points) and a percentage increase of 57%. Our final regression model predicts 25% of post-pandemic GAD-2 score variance (adjusted R^2^ = 0.25) (see Table 2). The final GAD-2 regression equation is: post-pandemic GAD-2 score = 0.50*pre-pandemic GAD-2 score + 0.14*(age × gender) + 0.12*(gender × child) + 1.55. That is, our model indicates that of the possible variables, the ones with a significance (*p* < 0.05) in predicting post-pandemic GAD-2 scores are pre-pandemic GAD-2 scores, the interaction between age and gender, and the interaction between gender and having to care for a child(ren) (see Table 3).

**Table 2:** GAD-2 regression model summary.

Model	R	R^2^	Adjusted R^2^	Standard error of the estimate
1	0.45^a^	0.21	0.21	1.68
2	0.49^b^	0.24	0.24	1.64
3	0.50^c^	0.25	0.25	1.63

^a^Predictors: (constant), Pre-GAD-2 sum.

^b^Predictors: (constant), Pre-GAD-2 sum, age x gender.

^c^Predictors: (constant), Pre-GAD-2 sum, age x gender, gender x child.

**Table 3:** GAD-2 regression coefficients.

Model		Unstandardized coefficients		Standardized coefficients	Significance	95% confidence interval for beta	
		Beta	Standard error	Beta	p-value	Lower bound	Upper bound
1	(Constant)	2.26	0.11		< 0.001	2.06	2.47
	Pre-GAD-2 sum	0.54	0.04	0.45	< 0.001	0.46	0.62
2	(Constant)	1.40	0.19		< 0.001	1.04	1.77
	Pre-GAD-2 sum	0.58	0.04	0.49	< 0.001	0.50	0.66
	Age × gender	0.01	0.00	0.19	< 0.001	0.01	0.02
3	(Constant)	1.45	0.19			1.09	1.81
	Pre-GAD-2 sum	0.59	0.04	0.50	< 0.001	0.51	0.67
	Age × gender	0.01	0.00	0.14	< 0.001	0.00	0.02
	Gender × child	0.25	0.08	0.12	0.003	0.09	0.42

For the conjunct variable of age and gender, our analysis indicates that an interaction between increasing age and non-man gender (that is, identifying as a woman or other gender) is associated with increased levels of post-pandemic GAD-2 scores. For the conjunct variable of gender and having a child(ren), our analysis indicates that the interaction between identifying as non-man in gender and having one or more children in the home is associated with increased levels of post-pandemic GAD-2 scores.

### Depressive symptoms

The mean pre-pandemic PHQ-9 score was 6.2 (95% CI 6.0 to 6.6) and increased to 10.8 (95% CI 10.4 to 11.1) following the pandemic (after March 11, 2020). This change represents a mean difference of 4.6 (out of a possible 27 points) and a percentage increase of 74%. Our final regression model predicts 32% of post-pandemic PHQ-9 score variance (adjusted R^2^ = 0.32) (see Table 4). The final PHQ-9 regression equation is: post-pandemic PHQ-9 score = 0.58*pre-pandemic PHQ-9 score + 0.10*(age × gender) + 0.09*(gender × child) + 3.92. That is, our model indicates that of the possible variables, the ones with a significance (*p* < 0.05) in predicting post-pandemic PHQ-9 scores are pre-pandemic PHQ-9 scores, the interaction between age and gender, and the interaction between gender and having to care for a child(ren) (see Table 5).

**Table 4:** PHQ-9 regression model summary.

Model	R	R^2^	Adjusted R^2^	Standard error of the estimate
1	0.55^a^	0.30	0.30	5.58
2	0.56^b^	0.32	0.32	5.50
3	0.57^c^	0.33	0.32	5.48

^a^Predictors: (constant), Pre-PHQ-9 sum.

^b^Predictors: (constant), Pre-PHQ-9 sum, age × gender.

^c^Predictors: (constant), Pre-PHQ-9 sum, age × gender, gender × child.

**Table 5:** PHQ-9 regression coefficients.

Model		Unstandardized coefficients		Standardized coefficients	Significance	95% confidence interval for beta	
		Beta	Standard error	Beta	p-value	Lower bound	Upper bound
1	(Constant)	6.00	0.35		< 0.001	5.32	6.68
	Pre-PHQ-9 sum	0.72	0.05	0.55	< 0.001	0.64	0.81
2	(Constant)	3.72	0.63			2.47	4.97
	Pre-PHQ-9 sum	0.76	0.05	0.57	< 0.001	0.67	0.84
	Age × gender	0.04	0.01	0.14	< 0.001	0.02	0.06
3	(Constant)	3.92	0.64			2.67	5.17
	Pre-PHQ-9 sum	0.76	0.05	0.58	< 0.001	0.68	0.85
	Age × gender	0.03	0.01	0.10	0.010	0.01	0.05
	Gender × child	0.72	0.29	0.09	0.014	0.15	1.29

For the conjunct variable of age and gender, our analysis indicates that an interaction between increasing age and non-man gender (that is, identifying as a woman or other gender) is associated with increased levels of post-pandemic PHQ-9 scores. For the conjunct variable of gender and having a child(ren), our analysis indicates that an interaction between identifying as non-man in gender and having one or more children in the home is associated with increased levels of post-pandemic PHQ-9 scores.

## Discussion

Drawing upon literature of what can impact mental health during the COVID-19 pandemic, we tested several variables that could contribute to post-pandemic levels of anxiety and depressive symptoms. Among the variables we tested were the intersectional variables of age × gender, age × marital status, gender × child (yes/no), gender × working location, and working location × child (yes/no). Of these variables, what we found to affect post-pandemic levels in GAD-2 and PHQ-9 scores were pre-pandemic GAD-2 and PHQ-9 scores, respectively, along with the intersection of age and gender and the intersection of gender and having a child(ren) in the home.

As mentioned, several other studies have found that women are experiencing greater levels of anxiety and depression following the COVID-19 pandemic^8–11^, which is consistent with non-man participants in our sample intersecting with age to lead to increased post-pandemic levels of GAD-2 and PHQ-9. However, current COVID-19 pandemic research mainly indicates decreasing levels of distress among older adults^9–13^. There are a few possible reasons for this finding. The distribution of age in our sample is skewed towards younger participants with GAD-2 participants having a mean age of 30.3 (± 13.3) years and PHQ-9 participants having a mean age of 30.4 (± 13.5) years. This is a limitation and could present a masking effect on the true impact of age in this conjunct variable. However, it is also possible that when age and gender intersect, rather than examining age in isolation, the effect of age on anxiety and depressive symptoms is seemingly the opposite of what it would be in isolation.

Working from home as it interacts with either gender or having a child(ren) was not a predictor of post-pandemic levels of anxiety or depressive symptoms, based on our findings. Although it may seem that working from home would contribute to poor mental health, our study indicates that this is not the case with anxiety or depression. A possible reason for this finding is that those working from home did not lose their jobs or livelihood due to the pandemic. Household income has consistently been identified as a social determinant of mental health with individuals from lower socio-economic status having a higher prevalence of anxiety and depressed mood^31^. Therefore, financial security and maintenance of family income or jobs could be facilitating better mental health outcomes following the COVID-19 pandemic. Other studies reinforce the results identified in this study that those who stopped working as a result of the pandemic have increased levels of distress^18,19^.

Although it may be expected that those parents working from home would have higher levels of mental distress during the pandemic, our findings indicated that working from home while having a child(ren) was not a predictor of either higher GAD-2 or PHQ-9 scores following the pandemic. There are several possible explanations for this finding, which future research could address. Our study did not look at the age of the child(ren). Having to care for a preschooler, supervise a child in elementary school, or having a high school student at home can be very different scenarios for a working parent. Further investigation could also explore how working from home with a child(ren) presents differently among those with varying degrees of support, such as having another caretaker in the home. Finally, a child’s personal needs can have varying impacts on a working parent. A study on parental burnout during the COVID-19 pandemic found increasing levels of burnout among parents who had children with special needs^20^.

It must be noted, other than pre-pandemic levels of GAD-2 and PHQ-9 scores, the two variables that have significance in predicting post-pandemic levels of anxiety and depressive symptoms are those that are intersectional with gender (age x gender and gender x child). In addition to having statistical significance, this finding has high clinical significance. Health care providers, especially mental health professionals, need to be aware and able to help women and those who identify as other genders (non-binary, agender, other) during and following the pandemic.

### Limitations

Two potential limitations of the study are recall bias and selection bias. Our study was designed as a cross-sectional survey that asked respondents to recall their pre-pandemic state to complete GAD-2 and PHQ-9. This method could have led to recall bias as respondents aimed to remember how they were feeling prior to the start of the pandemic. However, our study was conducted in the early months of the pandemic and asked respondents to recall only a few months earlier for their pre-pandemic scores.

A second limitation of the study was the risk for selection bias. Accessing the internet and use of technology may have excluded elders and skewed representation in the sample to a lower age. Additionally, those from lower socioeconomic groups and transient populations could be less represented due to barriers in internet access. However, the online design may have decreased social desirability bias and enabled respondents to freely and openly respond to sensitive survey questions.

## Conclusions

Our research has determined several factors that predict post-pandemic (following March 11, 2020) levels of anxiety and depressive symptoms among the general population, as measured through GAD-2 and PHQ-9. Namely, these factors are pre-pandemic levels of GAD-2 and PHQ-9 scores, for post-pandemic levels of GAD-2 and PHQ-9, respectively, along with the intersection of age and gender and gender and having a child(ren). Health care practitioners, especially those working in psychiatry and mental health, need to be aware of the extra burden of the pandemic on women and those who identify as other genders, especially those who are parents or care for children in their homes.

## Data Availability

The datasets used and analysed during the current study are available from the corresponding author on reasonable request.

## References

[1] Li Q, Guan X, Wu P, Wang X, Zhou L, Tong Y (2020). Early transmission dynamics in Wuhan, China, of novel coronavirus-infected pneumonia. N. Engl. J. Med..

[2] World Health Organization. *WHO Director-General’s Opening Remarks at the Media Briefing on COVID-19 - 11 March 2020*. https://www.who.int/director-general/speeches (2020).

[3] da Silva Junior FJG, Sales JCES, Monteiro CFDS, Costa APC, Campos LRB, Miranda PIG (2020). Impact of COVID-19 Pandemic on Mental Health of Young People and Adults: A Systematic Review Protocol of Observational Studies.

[4] Vigo D, Patten S, Pajer K, Krausz M, Taylor S, Rush B (2020). Mental Health of Communities during the COVID-19 Pandemic. Can. J. Psychiatry..

[5] Horiuchi S, Shinohara R, Otawa S, Akiyama Y, Ooka T, Kojima R (2020). Caregivers’ mental distress and child health during the COVID-19 outbreak in Japan. PLoS ONE.

[6] Jia R, Ayling K, Chalder T, Massey A, Broadbent E, Coupland C (2020). Mental health in the UK during the COVID-19 pandemic: cross-sectional analyses from a community cohort study. BMJ Open.

[7] Clinical Management [Internet]. https://apps.who.int/iris/bitstream/handle/10665/338871/WHO-2019-nCoV-clinical-web_annex-2021.1-eng.pdf. (2021).

[8] Wang C, Pan R, Wan X, Tan Y, Xu L, Ho CS (2020). Immediate psychological responses and associated factors during the initial stage of the 2019 coronavirus disease (COVID-19) epidemic among the general population in China. Int. J. Environ. Res. Public Health..

[9] Mazza C, Ricci E, Biondi S, Colasanti M, Ferracuti S, Napoli C (2020). A nationwide survey of psychological distress among Italian people during the covid-19 pandemic: Immediate psychological responses and associated factors. Int. J. Environ. Res. Public Health..

[10] Qiu J, Shen B, Zhao M, Wang Z, Xie B, Xu Y (2020). A nationwide survey of psychological distress among Chinese people in the COVID-19 epidemic: Implications and policy recommendations.

[11] Mental Health in Canada. *Covid-19 and Beyond CAMH Policy Advice*. (2020).

[12] Bulloch A, Zulyniak S, Williams J, Bajgai J, Bhattarai A, Dores A (2021). Poor mental health during the COVD-19 pandemic: Effect modification by age. Can. J. Psychiatry [Internet]..

[13] Bruine de Bruin W (2021). Age differences in COVID-19 risk perceptions and mental health: Evidence from a National U.S. Survey conducted in March. J. Gerontol. B Psychol. Sci. Soc. Sci..

[14] Shrestha, D.B., Thapa, B.B., Katuwal, N., Shrestha, B., Pant, C., & Basnet, B. *et al*. *Psychological Distress in Nepalese Residents During COVID-19 Pandemic: A Community Level Survey*. Vol. 20. (BMC Psychiatry. BioMed Central Ltd, 2020). 10.1186/s12888-020-02904-6 PMC7538049 33023563

[15] Mosheva M, Hertz-Palmor N, Dorman Ilan S, Matalon N, Pessach IM, Afek A (2020). Anxiety, pandemic-related stress and resilience among physicians during the COVID-19 pandemic. Depress. Anxiety.

[16] Ying Y, Ruan L, Kong F, Zhu B, Ji Y, Lou Z (2020). Mental health status among family members of health care workers in Ningbo, China, during the coronavirus disease 2019 (COVID-19) outbreak: A cross-sectional study. BMC Psychiatry.

[17] López-Núñez MI, Díaz-Morales JF, Aparicio-García ME (2021). Individual differences, personality, social, family and work variables on mental health during COVID-19 outbreak in Spain. Pers. Individ. Differ..

[18] Zhang SX, Wang Y, Rauch A, Wei F (2020). Unprecedented disruption of lives and work: Health, distress and life satisfaction of working adults in China one month into the COVID-19 outbreak. Psychiatry Res..

[19] Lawal AM, Alhassan EO, Mogaji HO, Odoh IM, Essien EA (2020). Differential effect of gender, marital status, religion, ethnicity, education and employment status on mental health during COVID-19 lockdown in Nigeria. Psychol. Health Med..

[20] Fontanesi L, Marchetti D, Mazza C, di Giandomenico SD, Roma P, Verrocchio MC (2020). The effect of the COVID-19 lockdown on parents: A call to adopt urgent measures. Psychol. Trauma Theory Res. Pract. Policy..

[21] Crenshaw, K. *Demarginalizing the Intersection of Race and Sex: A Black Feminist Critique of Antidiscrimination Doctrine, Feminist Theory and Antiracist Politics Recommended Citation Crenshaw, Kimberle () "Demarginalizing the Intersection of Race and Sex: A Black Feminist Critique of Antidiscrimination Doctrine, Feminist Theory and Antiracist Politics [Internet]*. Vol. 1989. http://chicagounbound.uchicago.edu/uclf. http://chicagounbound.uchicago.edu/uclf/vol1989/iss1/8 (University of Chicago Legal Forum).

[22] Kapilashrami A, Hankivsky O (2018). Intersectionality and why it matters to global health. Lancet.

[23] FagrellTrygg N, Gustafsson PE, Månsdotter A (2019). Languishing in the crossroad? A scoping review of intersectional inequalities in mental health. Int. J. Equity Health NLM (Medline).

[24] Eysenbach G (2004). Improving the quality of web surveys: The Checklist for Reporting Results of Internet E-Surveys (CHERRIES). J. Med. Internet Res. (JMIR Publications Inc.).

[25] Heidari S, Babor TF, de Castro P, Tort S, Curno M (2016). Sex and gender equity in research: Rationale for the SAGER guidelines and recommended use. Res. Integr. Peer Rev..

[26] Kroenke, K., Spitzer, R.L., Williams, J.B.W., Monahan, P.O., & Lö, B. *Anxiety Disorders in Primary Care: Prevalence, Impairment, Comorbidity, and Detection [Internet]*. https://www.annals.org (2017). 10.7326/0003-4819-146-5-200703060-00004 17339617

[27] Kroenke, K., Spitzer, R.L., & Williams, J.B.W. *The Patient Health Questionnaire-2 Validity of a Two-Item Depression Screener*. 10.1097/01.MLR.0000093487.78664.3C 14583691

[28] Staples LG, Dear BF, Gandy M, Fogliati V, Fogliati R, Karin E (2019). Psychometric properties and clinical utility of brief measures of depression, anxiety, and general distress: The PHQ-2, GAD-2, and K-6. Gen. Hosp. Psychiatry.

[29] Kroenke K, Spitzer RL (2002). Nursing & allied health database. Psychiatr. Ann..

[30] Kroenke, K., Spitzer, R.L., & Williams, J.B.W. *The PHQ-9 Validity of a Brief Depression Severity Measure*. 10.1046/j.1525-1497.2001.016009606.x PMC1495268 11556941

[31] Allen J, Balfour R, Bell R, Marmot M (2014). Social determinants of mental health. Int. Rev. Psychiatry.

